# Long-term survival after hepatic resection for colorectal liver metastases: a single-center study in Iran

**DOI:** 10.1186/s12893-024-02420-4

**Published:** 2024-05-03

**Authors:** Seyed Morteza Pourfaraji, Mehdi Nazari Moghadam, Ali Mohammad Moradi, Fatemeh Ojaghi Shirmard, Narjes Mohammadzadeh, Ali Jafarian

**Affiliations:** 1 School of Medicine, Tehran University of Medical Sciences, Tehran, Iran; 2 Division of Hepatopancreatobiliary and Liver Transplantation, School of Medicine, Imam Khomeini Hospital Complex, Tehran University of Medical Sciences, Tehran, Iran; 3 Department of Surgery, Imam Khomeini Hospital, Tehran University of Medical Sciences, Tehran, Iran

**Keywords:** Colorectal cancer, Liver metastases, Surgical resection

## Abstract

**Background:**

Surgical resection of colorectal cancer liver metastasis (CRLM) has been associated with improved survival in these patients. The purpose of this study was to investigate the usefulness of liver metastasectomy, also finding independent factors related to survival after liver metastasectomy.

**Methods:**

In a retrospective study, all patients with CRLM who underwent resection of liver metastases between 2012 and 2022 at Imam Khomeini Hospital Complex in Tehran, Iran, were enrolled. All patients were actively followed based on clinicopathologic and operative data.

**Results:**

A total of 248 patients with a median follow-up time of 46 months (Range, 12 to 122) were studied. Eighty-six patients (35.0%) underwent major hepatectomy, whereas 160 (65.0%) underwent minor hepatectomy. The median overall survival was 43 months (Range, 0 to 122 months), with estimated 1-, 3- and 5-year overall survival rates of 91%, 56%, and 42%, respectively. Multivariate analysis demonstrated that a metastasis size > 6 cm, major hepatectomy, rectum as the primary tumor site, and involved margin (< 1 mm) were independent factors associated with decreased overall survival (OS).

**Conclusion:**

Surgical resection is an effective treatment for patients with CRLM that is associated with relatively favorable survival. A negative margin of 1 mm seems to be sufficient for oncological resection.

## Introduction

Colorectal cancer (CRC) is a substantial public health concern in most parts of the world. In 2020, CRC emerged as the second leading cause of cancer-related death. (9.4% of all deaths due to cancer) and the third most frequently diagnosed malignancy (1.9 million new cases) worldwide [1]. Liver metastasis occurs in nearly 14–30% of patients at the first presentation of primary CRC (known as synchronous) [2–4]. Eventually, approximately 50% of patients with CRC will develop hepatic metastasis as their disease progresses [5].

Patients with hepatic metastases from CRC have a poor prognosis without therapeutic intervention. The median survival time ranges from 12 to 15 months, and survival rates are less than 5% in those patients [6]. Furthermore, curative metastasis resection is possible in only 20–30% of cases with CRLM [7]. Fortunately, patient eligibility for resection has increased, leading to an increase in the survival rate of CRLM patients in recent years with progress in surgical techniques, radiology, and systemic chemotherapy.

Despite the questions surrounding the selection criteria for curative resection in these patients, surgery remains the only way to achieve a cure (defined as more than ten years of survival) and the best treatment for increasing life expectancy in patients with colorectal liver metastases (CRLM). The 5-year survival rate is usually more than 50% for patients with resectable colorectal liver metastases (CRLM) who undergo metastasectomy [8]. Patients with small, metachronous tumors (metastases diagnosed after six months of primary tumor resection), and solitary metastases are more likely to be amenable to resection surgery [9]. Although many authors have identified prognosticators of survival in CRLM patients, the role of some variables in long-term survival has not been fully defined.

The primary goal of this study was to investigate our 10-year experience in metastasectomy of CRLM and demonstrate the long-term overall survival (OS) of patients in our tertiary care center. Additionally, we aimed to find independent variables that correlate with survival in patients with CRLM.

## Method

We performed a retrospective single-center study of all CRC patients with liver metastasis who underwent metastasectomy from 2012 to 2022 at the Imam Khomeini Hospital Complex, Tehran University of Medical Sciences, Tehran, Iran. The study was approved by the Research Ethics Committees of Imam Khomeini Hospital Complex in July 2023.

In our service, the decision to perform surgical resection for CRLM was based on the potential for complete removal of the tumor while ensuring an adequate Future liver remnant (FLR). The number and size of metastases were limiting factors when there was high concern about FLR. If the above-mentioned criteria were met, we operated on synchronous and metachronous metastases. In cases of synchronous CRLM after neoadjuvant chemotherapy, we preferred a liver-first surgery approach. We discussed chemo-unresponsive metastases in a hepatobiliary multidisciplinary team, and based on the disease progression and consensus of the MDT, we chose the most appropriate treatment. We considered the neoadjuvant chemotherapy for almost all synchronous CRLM. The exception was rare cases with one or two superficial CRLM that may be resected at colorectal surgery. The conversion criteria were to make the patient tumor-free and achieve adequate FLR after neoadjuvant chemo with or without portal vein embolization. In our center, first-line chemotherapy for metastatic CRC consists of fluorouracil-based regimens containing oxaliplatin, capecitabine, or a combination of both. XELOX (capecitabine and oxaliplatin) was mainly used as neoadjuvant systematic chemotherapy for our cases.

**Fig. 1:**
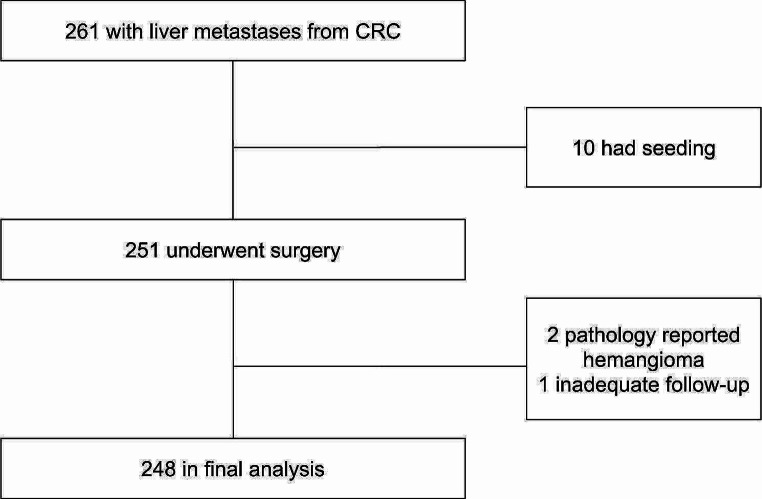
Patients included and excluded

Patients with metastatic seeding or hemangioma (based on a final pathology report of a resected tumor) were excluded (Fig. 1).

The following clinicopathological data were collected from pathology reports, operation notes, and archived files: age at diagnosis of the primary tumor, sex, location of CRC tumor (colon vs. rectum), presence of synchronous or metachronous metastases, number of metastases, largest diameter of hepatic metastases, liver steatosis grading based on the E Kleiner scoring system [10], type of hepatectomy (major for Sectionectomy, Hemihepatectomy, and Trisectionectomy versus minor for Partial or segmentectomy), surgical approach (‘colon’ first, ‘liver’ first and simultaneous), surgical margin status (R0 for margin ≥ 1 mm, and R1 for < 1 mm or involved tumor margin), tumor regression grade after chemotherapy [11], bilobar or ulilobar liver disease, status of lymphovascular and perineural invasion, metastatic tumor histological grade (poor, moderate, well-differentiated), and receiving neoadjuvant chemotherapy before liver surgery.

The main endpoint of this study was overall survival (OS), defined as the time from the date of the first liver surgery until the date of the last follow-up or death.

### Statistical analysis

Survival analysis was conducted using the Kaplan‒Meier method to estimate overall survival following the first metastasectomy in all patients. The Log rank test was used for limited variable. Univariate analysis via the Cox regression model with all factors was performed to determine the prognostic factors for OS. Age, sex, and all other variables with a P value < 0.100 were selected for further multivariate analysis. The Cox proportional hazard (CPH) model was accomplished for multivariate analysis to determine the independent and adjusted clinicopathological factors that impacted the overall survival of CRLM patients. All the statistical analyses were performed using STATA (version 17.0, Stata) and SPSS (version 27.0, IBM). P values < 0.05 in univariate and multivariate analyses were considered to indicate statistical significance.

## Results

### Patient demographic and surgical details

Overall, 248 patients who underwent liver metastasectomy for CRLM were included in our study. The median follow-up time for patients who were alive was 46 months (Range, 12 to 122). Most cases were male (*n* = 143, 57.7%), and the mean age at the time of liver resection was 54.27 years (SD = 11.00). The clinicopathological characteristics, patient demographics, and surgical details are shown in Table 1. The primary cancer location was the colon in 138 (55.4%) patients and the rectum in 111 (44.6%) patients. Most patients (*n* = 168, 69.4%) had synchronous CRLM, while 74 (30.6%) had metachronous CRLM. The majority of the subjects (*n* = 217, 87.9%) received neoadjuvant chemotherapy before metastasectomy. Eighty-six patients (35.0%) underwent major hepatectomy, whereas 160 (65.0%) underwent minor hepatectomy. The surgical approaches used in the synchronous metastasis group were colon-first (*n* = 79, 48.5%), liver-first (*n* = 72, 44.2%), and simultaneous (*n* = 12, 7.4%). Liver involvement mainly was mainly unilobar (*n* = 171, 69.5%), and bilobar involvement occurred in 75 (30.5%) patients.

**Table 1:** Patients demographics

Variable	N^1^ (%)
Age, years (mean)	54.27
Sex	
Female	105 (42.3%)
Male	143(57.7%)
Primary tumor location	
Colon	138 (55.4%)
Rectum	111(44.6%)
chronology	
synchronous	168 (69.4%)
Metachronous	74 (30.6%)
Neoadjuvant therapy	
No	30 (12.1%)
Yes	217 (87.9%)
Approach of surgery (just for synchronous)	
Colon first	79 (48.5%)
Liver first	72 (44.2%)
simultaneously	12 (7.4%)
Grade of tumor	
1 (well)	49 (26.1%)
2 (moderate)	137 (72.9%)
3 (poor)	2 (1.1%)
Hepatectomy	
Minor	160 (65.0%)
Major	86 (35.0%)
liver steatosis grade	
0	98 (50%)
1	68 (34.7%)
2	24 (12.2%)
3	6 (3.1%)
Lymphovascular Invasion	
No	31 (16.6%)
Yes	156 (83.4%)
Perineural Invasion	
No	155 (83.3%)
Yes	31 (16.7%)
Tumor Regression Grade response	
Poor	26 (14.2%)
Partial	138 (75.4%)
Complete	19 (10.4%)
Resection	
R0	190 (78.2%)
R1	53 (21.8%)
Size of metastases	
< 6 cm	180 (75.6%)
≥ 6 cm	58 (24.4%)
Surgical margin	
1 mm	31 (12.8%)
> 1 mm	159 (65.4%)
< 1 mm	53 (21.8%)
Lobar involvement	
Unilobar	171 (69.5%)
Bilobar	75 (30.5%)
Survival	
Death	129 (52%)
Alive	119 (48%)
Mortality (In-hospital – 30 days)	6 (2.4%)

^1^ The number of participants with available data

### Pathology

The mean largest tumor size was 44.3 mm (SD = 32.3), and the mean number of metastases was 1.89 (SD = 1.29) according to the pathology reports. Lymphovascular invasion (LVI) and Perineural invasion (PNI) were reported for 187 (75%) patients. Among these patients, LVI was present in 156 (83.4%), while PNI was found in 31 (16.7%) patients.

## Survival

**Fig. 2:**
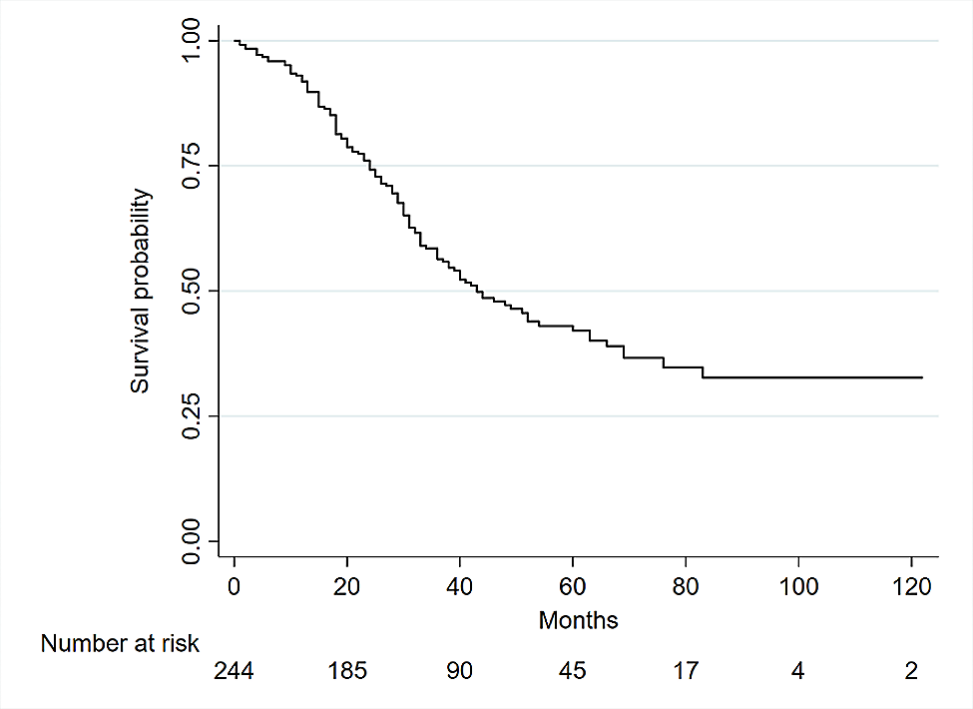
Kaplan‒Meier curve for overall survival

**Fig. 3:**
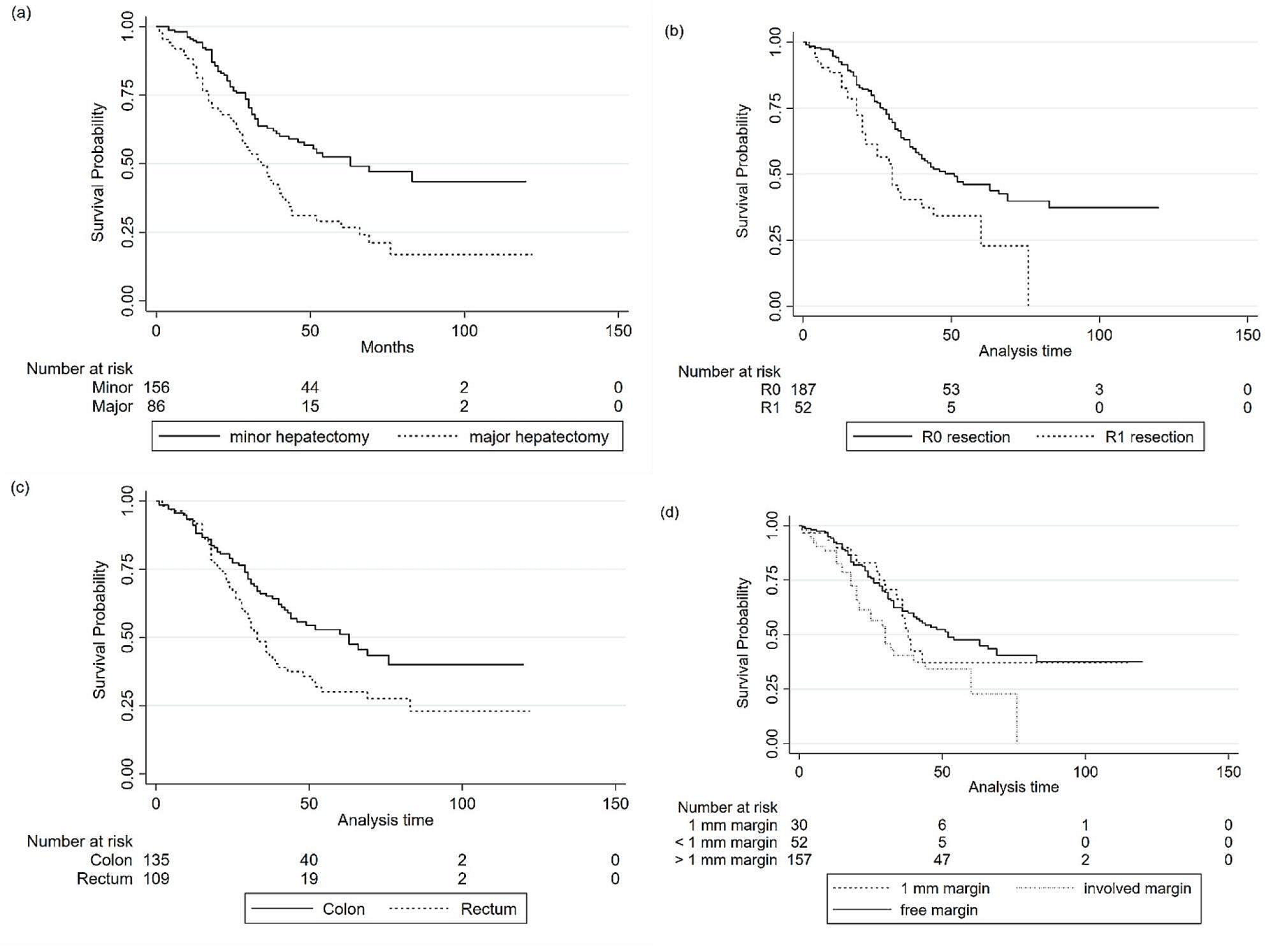
Survival curves based on the independent prognostic factors. (**a**) overall survival of patients who underwent minor hepatectomy versus major hepatectomy (**b**) overall survival of patients who underwent R0 resection versus R1 resection (**c**) overall survival of patients with colon as the primary site versus rectum (**d**) overall survival of patients based on surgical margins

Six patients died within the first 30 days after metastasectomy, for an operation mortality rate of only 2.4%. The median overall survival was 43 months (Range, 0 to 122 months), with estimated 1-, 3- and 5-year overall survival rates of 91%, 56%, and 42%, respectively. The survival curve is shown in Fig. 2. The median survival of cases with surgical margins less than 1 mm (30 months, range 0 to 76) was statistically (log rank, P value = 0.003) lower than participants with higher than 1 mm surgery margins (52 months, range 0 to 120). However, the difference between the median survival of patients with a 1 mm margin (38 months, range 0 to 116) was not statistically (P value = 0.554) lower than cases with free margins (See Fig. 3).

Univariate (Cox regression) and multivariate (Cox proportional hazards model) analyses were performed to determine the clinicopathological variables correlated with OS after metastasectomy (Table 2).

**Table 2:** Prognosticator of overall survival (univariate and multivariate analysis)

Variable	Univariate analysis		Multivariate analysis	
	P value	HR (CI)	P value	HR adjusted (CI)
Age				
< 65 years	Ref ^1^	NA	NA	NA
≥ 65 years	0.860	1.041 (0.663–1.635)	0.350	1.251 (0.782–2.001)
Sex				
Female	Ref	NA	NA	NA
Male	0.395	0.859 (0.607–1.218)	0.232	0.802 (0.558–1.152)
Chronology				
Synchronous	Ref	NA	NA	NA
Metachronous	0.512	1.134 (0.779–1.649)	NA	NA
Neoadjuvant therapy				
No	Ref	NA	NA	NA
Yes	0.715	0.911 (0.553–1.501)	NA	NA
Number of liver metastases				
< 3	Ref	NA	NA	NA
≥ 3	**0.015**	1.608 (1.097–2.357)	0.384	1.205 (0.792–1.832)
Largest metastasis size				
<6 cm	Ref	NA	NA	NA
≥ 6 cm	**0.008**	1.694 (1.151–2.493)	**0.052**	1.536 (0.997–2.367)
Hepatectomy				
Minor	Ref	NA	NA	NA
Major	**0.0005**	1.850 (1.304–2.625)	**0.014**	1.666 (1.107–2.508)
LVI				
No	Ref	NA	NA	NA
Yes	**0.011**	2.134 (1.188–3.834)	NA	NA
PNI				
No	Ref	NA	NA	NA
Yes	0.271	1.333 (0.799–2.224)	NA	NA
liver steatosis grade				
0	Ref	NA	NA	NA
1	**0.009**	0.547 (0.347–0.862)	NA	NA
2	0.290	0.706 (0.370–1.347)	NA	NA
3	0.672	0.802 (0.290–2.220)	NA	NA
Grade of tumor (differentiation)				
Well	Ref	NA	NA	NA
Moderate	0.686	1.093 (0.711–1.680)	NA	NA
Poor	**0.003**	9.793 (2.221–43.190)	NA	NA
Tumor location				
Colon	Ref	NA	NA	NA
Rectum	**0.006**	1.626 (1.149–2.301)	**0.0001**	2.175 (1.458–3.243)
Approach of surgery (just for synchronous)				
Colon first	Ref	NA	NA	NA
Liver first	0.071	1.522 (0.965–2.399)	NA	NA
simultaneously	0.477	0.688 (0.245–1.931)	NA	NA
Resection				
R0	Ref	NA	NA	NA
R1	**0.005**	1.793 (1.194–2.691)	**0.05**	1.567 (1.001–2.454)
Surgical margin				
> 1 mm	Ref		NA	NA
1 mm	0.564	1.172 (0.684–2.009)	NA	NA
< 1 mm	**0.004**	1.840 (1.213–2.792)	NA	NA
TRG				
Poor	Ref	NA	NA	NA
Partial	0.663	NA	NA	NA
Complete	0.343	NA	NA	NA
Time between primary tumor to metastases (for metachronous)				
≤ 12 months	Ref	NA	NA	NA
>12 months	0.765	1.143 (0.477–2.736)	NA	NA
Lobar involvement				
Unilobar	Ref	NA	NA	NA
Bilobular	0.319	1.219 (0.833–1.754)	NA	NA

^1^ reference group

Bold values indicate significance at p < 0.05

The univariate analysis suggested that the following factors were significantly associated with worse OS: more than two liver metastases (HR = 1.608, 95% CI, 1.097–2.357; *P* = 0.015), largest metastases size > 6 cm (HR = 1.694, 95% CI, 1.151–2.493; *P* = 0.008), rectum as a location of the primary tumor (HR = 1.626, 95% CI, 1.149–2.301; *P* = 0.006), major hepatectomy (HR = 1.850, 95% CI, 1.304–2.625; P = < 0.001), R1 resection (HR = 1.793, 95% CI, 1.194–2.691; *P* = 0.005), and positive LVI (HR = 2.134, 95% CI, 1.188–3.834; *P* = 0.011). Additionally, poorly differentiated tumors were associated with worse OS (HR = 9.793, 95% CI, 2.221–43.190; *P* = 0.003). However, the limited number of patients in this tumor differentiation category (*N* = 2) led to a hazard ratio with a broad CI. Further multivariate analysis was conducted for several variables, including age, sex, number of liver metastases (< 3, ≥ 3), largest metastasis size (< 6 cm, ≥ 6 cm), type of hepatectomy, location of the primary tumor, and resection status (R0 vs. R1). Multivariate analysis demonstrated that a metastasis size > 6 cm, major hepatectomy, R1 resection, and rectum as the primary tumor site were independent factors associated with worse OS. (Fig. 3)

## Discussion

Due to improvements in surgical techniques, imaging, and active systemic treatment CRLM has changed from a fatal disease to a manageable condition with a 5-year survival rate of more than 50% in some studies [12]. The median survival of CRLM patients who underwent curative surgery ranged from 1.7 to 7.3 years [13]. Our data demonstrated 1-year, 3-year, and 5-year survival rates of 91%, 56% and 42%, respectively. Additionally, the operative mortality rate was only 2.4% in our series. Therefore, our series confirmed hepatic resection as a relatively safe and effective treatment for selected patients with CRLM. Our results are in keeping with the literature [14, 15].

In the past, a resection margin width of at least 10 mm was considered the minimum width needed to achieve a better prognosis [16–19]. As a result of the development of systematic chemotherapy, many studies have revealed the trend of using a narrower width as an adequate free margin [20, 21]. According to our analysis, the median survival time of patients with R1 resection was 18 months shorter than cases with R0 resection (30 vs. 48 months), and univariate analysis revealed the margin as a significant predictor. Further analyses showed that the median survival of patients with 1 mm margins was not statistically lower than that of cases with higher than 1 mm margins. Also, the Cox regression model did not demonstrate that the “1 mm margin” was not a significant prognostic factor for poor survival. Similarly, multiple studies reported these findings and confirmed that a margin greater than 1 mm is enough for more prolonged survival and better outcomes [22, 23].

The impact of the interval between the primary tumor and liver metastasis on OS is unclear. Noémi Reboux et al. reported a significantly better OS in the metachronous group based on a population-based study of 26,813 patients [24]. However, in accordance with the findings of several articles, we did not observe significant differences in survival between patients with synchronous and metachronous metastasis [25–28].

Although there is a discrepancy in determining the optimal cutoff for metastasis size, multiple studies have shown that a larger metastasis size is a negative predictor of OS [4, 13]. Some clinical risk scoring systems recommend using 5 cm as a cutoff [29, 30]; however, we found that 6 cm was the optimal cutoff for predicting long-term survival in our patients. The receiver operating characteristic (ROC) curve was used to determine the ideal cutoff in our series. Multivariate analysis demonstrated that a tumor diameter greater than 6 cm significantly decreased OS.

In many previous studies, the survival of patients with liver metastases of right colon cancer, including those of the rectum, has been reported to be worse than that of patients with liver metastases of the left colon [25, 31]. However, this study showed that the OS after resection of rectal CRLMs was significantly poorer.

The type of hepatectomy was an independent factor for long-term OS. In the current analysis, major hepatectomy was significantly associated with a worse prognosis. As per our center’s protocol, it is recommended that patients with synchronous CRLM and primary unresectable cases undergo neoadjuvant chemotherapy to reduce the size of the metastases and facilitate the preservation of a suitable future liver remnant. The current study did not demonstrate a statistically significant correlation between the administration of neoadjuvant chemotherapy and overall survival. This result is in agreement with previously published studies [32, 33].

The resectability status is well-defined for certain types of cancer, such as pancreatic cancer [34]. However, the definitions of primary resectable, borderline resectable, and primary unresectable for CRLM are not clearly explained by researchers. The introduction of the idea of borderline tumor for CRLM was proposed in 2007 by Jean-Nicolas Vauthey due to heterogeneity in long-term prognosis, particularly in cases with R1 resection or extrahepatic diseases [35]. In the last decade, authors suggested various definitions of CRLM borderline tumors [36–38]. Almost all presented definitions were based on the difficulty of achieving R0 resection or the high malignancy of tumors (larger or more metastases, higher level of CEA, …) [39]. Based on literature and clinical experience, borderline resectable cases are defined as those that may not become tumor-free or patients with inadequate (< 20%) FLR after resection.

In our analysis, we investigated the associations between pathological indexes of resected liver tissue, including tumor regression grade (TRG), perineural invasion (PNI), and lymphovascular invasion (LVI), and long-term survival. The lymphovascular invasion rate was greater in our cohort (83.4%) than in other similar studies [40, 41]. We hypothesize that this observed variation can be attributed to two main reasons: First, our hospital is a tertiary referral center, so we usually manage more advanced patients; second, our pathology department analyzes more tissue blocks, which is significantly associated with a higher probability of diagnosis of lymphovascular invasion. Our analysis revealed LVI as an independent adverse prognostic factor. Sakamoto et al., based on the meta-analysis of multiple studies, reported the same results and confirmed that the presence of LVI in liver metastasis significantly and negatively affects patient prognosis [42]. However, our findings did not confirm the presence of the PNI or TRG as significant negative factors for OS.

Although, as in recent studies, hepatic steatosis does not affect the survival of CRLM patients [43], in our series, mild steatosis in the remaining underlying liver, compared to higher degrees of steatosis, was associated with positive effects on survival in patients.

One of the limitations of this study is its retrospective nature, which caused some missing data, especially in pathological reports and information on primary colorectal cancer. Furthermore, our hospital is a referral hepatobiliary center in Iran, so primary systematic therapy and colorectal cancer resection of primary tumors from some patients have previously been performed at other centers. For this reason, it was not feasible to use the same protocol for the surgical and oncological approach for all the patients.

## Conclusion

Our data, which support the findings of previous studies, suggest that liver metastasectomy is associated with a reasonable overall survival rate in patients with CRLM. The resection margin and volume of liver disease (number and size of lesions) were factors affecting OS in our study. A margin of 1 mm is sufficient for liver metastasectomy, and we suggest that patients with CRLM be presented to the hepatobiliary multidisciplinary team so that the best treatment approach for every individual patient can be proposed by considering all radiological, oncological, and surgical aspects.

## Data Availability

The datasets used and/or analyzed during the current study are available from the corresponding author on reasonable request.

## Abbreviations

CRC: colorectal cancer
CRLM: colorectal cancer with liver metastasis
OS: overall survival
CI: confidence interval
LVI: lymphovascular invasion
TRG: tumor regression grade
PNI: perineural invasion
